# Identification of prefrontal cortex protein alterations in Alzheimer’s disease

**DOI:** 10.18632/oncotarget.24303

**Published:** 2018-01-24

**Authors:** Maria Garranzo-Asensio, Pablo San Segundo-Acosta, Javier Martínez-Useros, Ana Montero-Calle, María Jesús Fernández-Aceñero, Anna Häggmark-Månberg, Alberto Pelaez-Garcia, Mayte Villalba, Alberto Rabano, Peter Nilsson, Rodrigo Barderas

**Affiliations:** ^1^ Biochemistry and Molecular Biology Department I, Chemistry Faculty, Complutense University of Madrid, Madrid, Spain; ^2^ Translational Oncology Division, OncoHealth Institute, Fundacion Jimenez Diaz University Hospital, Madrid, Spain; ^3^ Servicio de Anatomía Patológica Hospital Clínico San Carlos, Instituto de Investigación Sanitaria del Hospital Clínico San Carlos (IdISSC), Departamento de Anatomía Patològica, Facultad de Medicina, Complutense University of Madrid, Madrid, Spain; ^4^ Affinity Proteomics, SciLifeLab, School of Biotechnology, KTH – Royal Institute of Technology, Stockholm, Sweden; ^5^ Department of Pathology, Hospital Universitario La Paz, IdiPAZ, Madrid, Spain; ^6^ Alzheimer Disease Research Unit, CIEN Foundation, Queen Sofia Foundation Alzheimer Center, Madrid, Spain; ^7^ UFIEC, National Institute of Health Carlos III, Majadahonda, Madrid, Spain

**Keywords:** Alzheimer’s disease, proteomics, neurodegeneration, protein/antibody microarrays, Gerotarget/Aging

## Abstract

Alzheimer’s disease (AD) is the most common form of dementia in developed countries. A better understanding of the events taking place at the molecular level would help to identify novel protein alterations, which might be used in diagnosis or for treatment development. In this study, we have performed the high-throughput analysis of 706 molecules mostly implicated in cell-cell communication and cell signaling processes by using two antibody microarray platforms.

We screened three AD pathological groups -each one containing four pooled samples- from Braak stages IV, V and VI, and three control groups from two healthy subjects, five frontotemporal and two vascular dementia patients onto Panorama and L-Series antibody microarrays to identify AD-specific alterations not common to other dementias. Forty altered proteins between control and AD groups were detected, and validated by i) meta-analysis of mRNA alterations, ii) WB, and iii) FISH and IHC using an AD-specific tissue microarray containing 44 samples from AD patients at different Braak stages, and frontotemporal and vascular dementia patients and healthy individuals as controls.

We identified altered proteins in AD not common to other dementias like the E3 ubiquitin-protein ligase TOPORS, Layilin and MICB, and validated the association to AD of the previously controverted proteins DDIT3 and the E3 ubiquitin-protein ligase XIAP. These altered proteins constitute interesting targets for further immunological analyses using sera, plasma and CSF to identify AD blood- or cerebrospinal fluid-biomarkers and to perform functional analysis to determine their specific role in AD, and their usefulness as potential therapeutic targets of intervention.

## INTRODUCTION

Alzheimer’s disease (AD) is the most common form of dementia in developed countries. This progressive and fatal illness manifests itself by cognitive and memory deterioration and involves a huge burden on daily life [1]. AD pathological hallmarks are characterized by the aggregation and subsequent formation of amyloid β plaques, tau hyperphosphorylation and tangle formation, inflammation, and oxidative stress; all of which contribute to structural and functional neuronal loss [2].

Despite the extensive research focused on the analysis of AD, the exact mechanisms triggering the disease that should produce massive proteome changes are still unclear. In addition, up to date, there is no specific reliable biomarker for AD, and the only definite diagnosis is made through *post mortem* neuropathology [3–5]. In cerebrospinal fluid (CSF), the measurement of Aβ42, total tau, and hyperphosphorylated tau are the most commonly used diagnostic tools [4]. However, even though the assays have high specificity and sensitivity, their positive predictive value is low for prodromal AD, which makes them useful only to help corroborating a possible AD diagnosis of patients that exhibit cognitive deficits but not for the detection of patients in the early stages of the disease [5]. Therefore, the identification of new AD-specific altered proteins, complexes and pathways as markers of the disease would be useful for diagnosis, prognosis and management of AD patients, to differentiate AD from other forms of dementia, and might help to find new targets for treatment development.

Several proteomics approaches have been tried to get further insights into pathways and molecular changes related to the disease to identify AD markers [6–9], with most of the studies focused on the analysis of AD patients *vs* healthy controls using CSF, serum or plasma [8, 10, 11]. Most proteomics studies are based on the use of mass-spectrometry due mainly to the increase in the resolution of the mass spectrometers [12]. However, despite the fact that mass-spectrometry driven proteomics has many advantages; it also presents one main caveat, which is its limited sensitivity in complex samples for most of the signaling-associated proteins (i.e. cytokines, growth factors, proteins implicated in cell cycle…). These proteins are present at so very low concentrations that are barely detected by mass spectrometry [13–15]. Therefore, antibody driven proteomics remain as the main solution for the high-throughput analysis of those molecules that are implicated in cell-cell communication and cell signaling processes, since they reach higher sensitivity. Therefore, antibody microarrays offer such a combination of sensitivity, and cost-effective multiplexing capabilities that makes them an affordable strategy for AD screening of alterations and biomarker identification [15, 16].

AD related changes along the progression of the disease selectively involve the transentorhinal cortex, spreading to the rest of the limbic system, and finally, to the more diffuse affectation of the neocortex [17]. Therefore, the analysis of the different brain sections might help decipher the massive proteome alterations occurring in AD as a consequence of the progression of the disease, get further insights into the AD pathophysiology and help to identify targets and pathways underlying particular clinical behavior of AD patients. In this study, we have used antibody microarray-based quantitative proteomics for the analysis of 706 signaling molecules as a platform to increase the knowledge on the pathophysiological mechanisms altered during the progression of AD. We have focused on the prefrontal cortex of AD patients and controls to identify AD-specific alterations non-related to other dementias and alterations related to AD progression. Forty altered proteins between controls and AD cases were detected, pointing out to AD-specific pathways deregulation, protein interaction networks, and potential novel targets of intervention. Protein deregulation was validated by i) meta-analysis of mRNA alterations from transcriptomic studies, ii) WB using AD and control samples, and iii) immunohistochemistry (IHC) and fluorescence *in situ* hybridization (FISH) using an AD-specific tissue microarray (TMA). Validated data provide novel information in the pathology. In addition, we observed by meta-analysis the complementarity of the antibody microarray data with that derived from mass-spectrometry based proteomics studies for the identification of protein alterations in AD. Identified altered proteins constitute interesting candidates to be further analyzed using sera, plasma and CSF to determine their usefulness as blood- or CSF- diagnostic biomarkers and to perform functional assays to help in the discovery of novel AD-specific therapeutic markers.

## RESULTS

**Figure 1:**
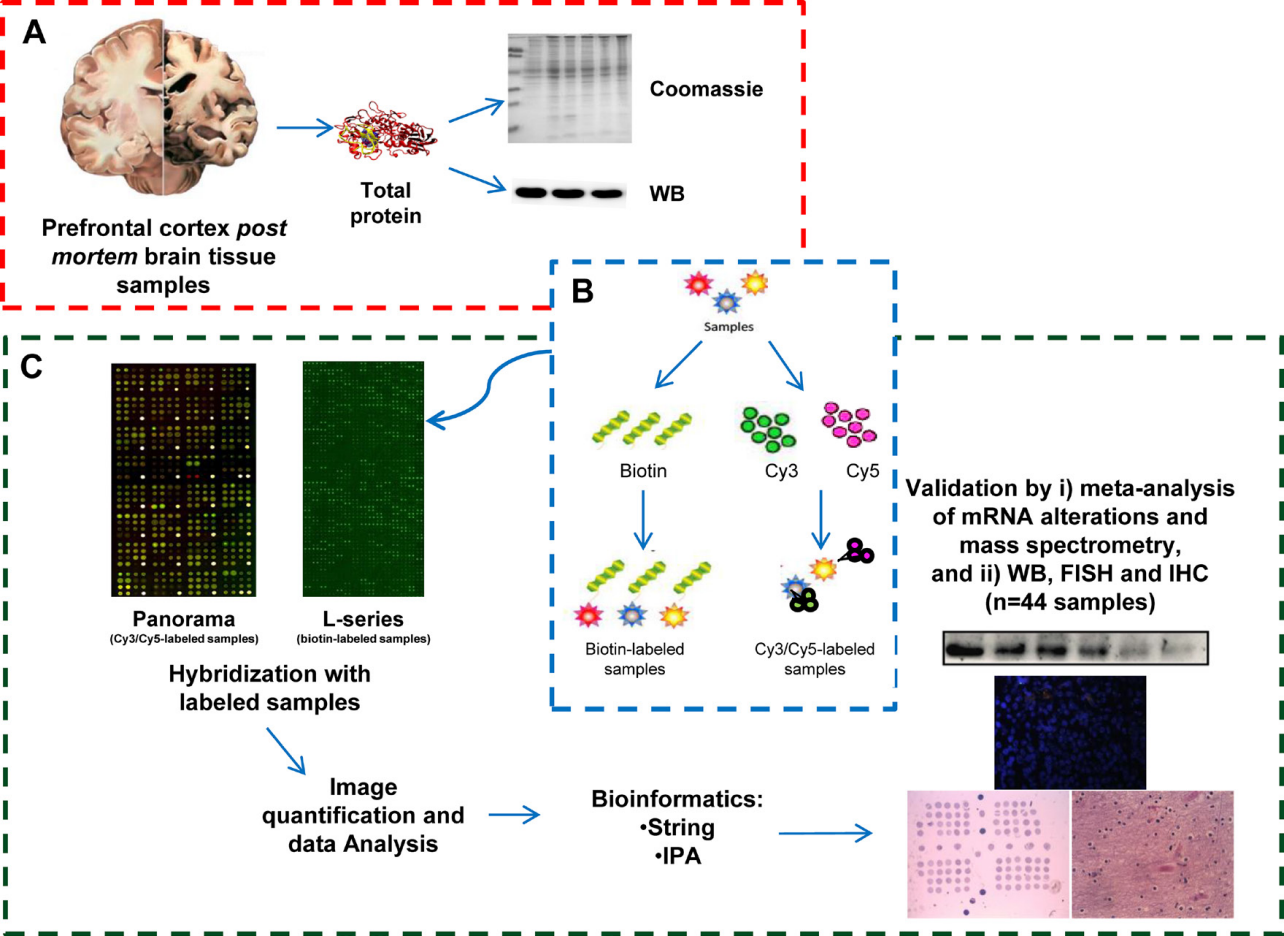
Schematic representation of the identification of AD-specific protein alterations non-related to other dementias by high-density antibody microarray (**A**) Protein Samples and extraction. Prefrontal cortex brain tissue protein extracts from: i) healthy individuals, ii) Vascular Dementia, and iii) Frontotemporal Dementia patients as controls of the study, iv) Braak IV, v) Braak V, and vi) Braak VI from AD patients as pathological groups of the study were obtained by mechanical disaggregation. After protein quantification, the quality of the protein extracts was assessed by Coomassie Blue staining and WB analysis with an anti-tubulin antibody as loading control. (**B**) Sample labeling. Pooled protein extracts of the indicated group conditions were either labeled with Cy3 dye or Cy5 dye, and biotin. (**C**) Microarray hybridization, image quantification, data analysis and validation of the results. Microarray hybridization was performed according to the recommendation of the manufacturers. Panorama antibody microarrays were probed simultaneously with one control group and one AD pathological group labeled with Cy3 and Cy5 or *vice versa*. RayBio Label-Based Human Antibody Arrays 493 antibody microarrays were separately incubated using one array per condition with biotin-labeled samples followed by Streptavidin-Cy3 to detect bound proteins captured by the antibodies. After image quantification with GenePix Pro7.1, normalization and data analysis, those proteins significantly altered in AD in comparison to all controls (healthy individuals, FTD and VD patients) were further validated by meta-analysis of large post-mortem studies examining the mRNA expression levels in prefrontal cortex tissue from late-onset AD and control patients, and other neurodegenerative conditions, or mass-spectrometry studies, and by here performed WB, and FISH and IHC with an AD-specific TMA using a large cohort of samples.

In the present study, we have performed the profiling of protein alterations in the prefrontal cortex of AD patients in comparison to healthy individuals and patients with other dementias as controls by antibody microarrays-driven proteomics to identify specific deregulated proteins associated to AD and non-related to other dementias, and potential intermediate to advance markers of AD progression. A schematic representation of the work-flow of the study, from protein extraction, screening of the antibody microarrays and data analysis, to validation of the protein alterations by i) meta-analysis of mRNA alterations from transcriptomic studies and meta-analysis of mass-spectrometry proteomics studies, and ii) here performed WB, IHC and FISH, is depicted in Figure 1.

In total, we have analyzed six different experimental groups using probe-directed antibody microarrays for the profiling of alterations in 706 immunomodulators, cytokines, chemokines, adipocytokines, growth factors, proteases and other proteins involved in cell signaling processes. For antibody microarrays experiments, we have pooled well-characterized samples from the prefrontal cortex of individuals with a common pathologic phenotype: three different AD groups corresponding to Braak IV, Braak V and Braak VI, and three control groups corresponding to frontotemporal dementia patients (FTD), vascular dementia patients (VD) and healthy individuals. We analyzed pooled samples from each group to avoid potential aberrations and biological variations appearing in individual samples, which might increase the capacity to identify the most significant and consistent changes between pooled samples from well-characterized AD, FTD, and VD patients and healthy individuals.

### Profiling of Alzheimer’s disease protein alterations with panorama and L-series antibody microarrays

Soluble protein contents from the prefrontal cortex tissue from the brain of *post mortem* individuals diagnosed with AD at Braak stages IV, V and VI as pathological groups and FTD and VD patients and healthy individuals as control groups were obtained, trying to match as much as possible the age and gender of the individuals in each group (Table 1).

**Table 1:** Sociodemographic and neuropathological data of the subjects analyzed in the antibody microarrays

			Gender		
Group	Condition^^^	Sample number	Male	Female	Age (years ± SD)^*^
Control	Healthy	177, 279	–	2	78 ± 28
Control	Fronto Temporal Dementia (FTD)	52, 54, 75, 119, 200	2	3	69 ± 11
Control	Vascular Dementia (VD)	8, 53	1	1	90 ± 4
AD (Braak IV)	AD Intermediate Stages	13, 67, 72, 143	2	2	88 ± 6
AD (Braak V)	AD Advanced Stage I	22, 31, 73, 106	2	2	86 ± 3
AD (Braak VI)	AD Advanced Stage II	27, 32, 36, 37	2	2	85 ± 5

^^^The pathophysiological assessment was performed by immunohistochemistry at the BT-CIEN Tissue Bank of the Spanish Research Center for Neurological Diseases Foundation (CIEN Foundation) using the prefrontal cortex of the brain tissue of AD patients and controls [1, 89, 90]. ^*^Age is given in mean (in years) of the samples. SD, Standard Deviation.

**Figure 2:**
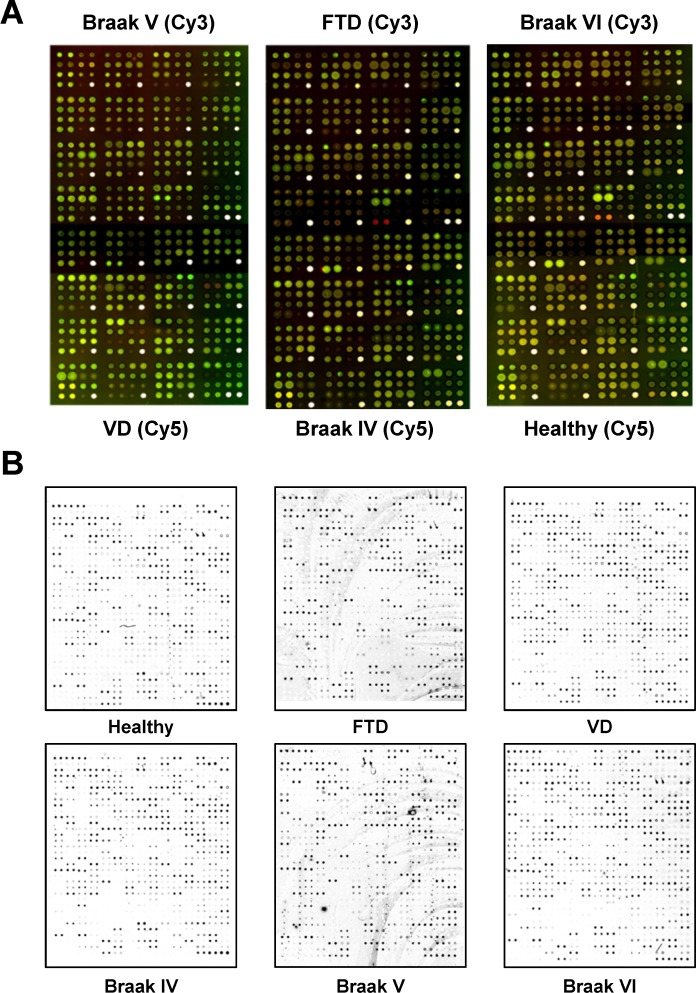
Representative images of the antibody microarrays used in the study to identify AD-specific protein alterations non-related to other dementias (**A**) The combined images corresponding to the two fluorescence emission wavelengths of Panorama antibody microarrays incubated with equals amounts of an AD pathological group and a control group alternatively labeled with Cy3 and Cy5 to avoid any concerns related to the labeling of the samples with specific dyes. (**B**) Individual performance of RayBio Label-Based Human Antibody Arrays 493 antibody microarrays incubated separately with the indicated pooled biotin-labeled group condition followed by incubation with Cy3-streptavidin.

For Panorama antibody microarrays, equal amounts of Cy3- and Cy5-labeled extracts from one of the three pathological AD groups and one of the three control groups (5 μg/ml) were incubated on each array to identify alterations in molecules implicated in cell signaling (Figure 2A). We next investigated for alterations in the expression of immunomodulators, cytokines, chemokines, adipocytokines, growth factors and proteases using the L-series antibody microarrays. The same experimental groups and tissue samples than above but labeled with biotin were used. Representative images of the indicated analyzed conditions followed by incubation with Cy3-streptavidin are shown in Figure 2B. The dynamic range of the intensity response -3 orders of magnitude for both series antibody microarrays-, and the signal to noise ratios ≥1.9 for both antibody microarrays, showed typical values of fluorescent-based antibody microarrays [18]. In addition, the morphology of the spots, and the positive and negative controls, which showed consistent correctness, were checked for both antibody microarray platforms.

To cope with potential concerns related to intra-array and interarray reproducibility, we first analyzed spot duplicates in both microarrays and observed a good correlation among fluorescence intensity of duplicates in both array types (Supplementary Figure 1). Then, we performed a dye swapping experiment in the Panorama antibody microarrays since samples are differently labeled with Cy3 or Cy5 (Supplementary Figure 1), in contrast to L-series microarrays where samples are only labeled with biotin. A good correlation among the fluorescence intensities of the proteins labeled with different dyes was also determined. Collectively, these analyses overcome potential concerns about the performance of the antibody microarrays.

### Differential protein expression analysis in Alzheimer’s disease

Then, we investigated for altered up- or down-regulated protein expression in AD in comparison to the control group to minimize any bias in the pooling strategy because of a low number of samples in any group. We calculated the protein expression AD/Control ratio using the median normalized fluorescence intensities obtained from the microarrays, considering relevant those proteins with at least ratios ≥1.5 or ≤0.67, setting this cut-off as previously done [19–21].

We identified a total of 6 altered proteins from the Panorama antibody microarrays and 34 from the L-series antibody microarrays (Table 2). The 63.5% of the deregulated proteins, including immunomodulators, cytokines, receptors, growth factors or proteases, showed up-regulation in AD in comparison to controls. From the total of the 40 deregulated proteins, we observed that 15 proteins were associated with the immune system, highlighting the important role of the inflammatory response in the AD pathogenesis [22, 23].

**Table 2:** Proteins significantly differentially expressed in AD in comparison to control groups identified with antibody microarrays

Protein name	Gene name	Uniprot ID	MFI ± SD^*^ AD groups	MFI ± SD^*^ Control groups	AD/Control Ratio^^^
Transcription factor GATA-4	GATA4	P43694	659 ± 241	127 ± 294	5.16
Calcitonin	CALCA	P01258	663 ± 439	222 ± 453	2.98
Chromogranin-A	CHGA	P10645	200 ± 71	79 ± 36	2.52
Mannose-binding protein C	MBL2	P11226	178 ± 87	75 ± 53	2.36
Ferritin Light Chain	FTL	P02792	1351 ± 383	580 ± 930	2.33
Ephrin type-A receptor 6	EPHA6	Q9UF33	160 ± 87	72 ± 53	2.21
MHC class I polypeptide-related sequence B	MICB	Q29980	135 ± 78	62 ± 50	2.15
Annexin A7	ANXA7	P20073	182 ± 96	93 ± 16	1.97
Interleukin-34	IL34	Q6ZMJ4	240 ± 112	128 ± 49	1.87
Galectin-3-binding protein	LGALS3BP	P17931	148 ± 67	79 ± 247	1.86
Kallikrein-8	KLK8	O60259	146 ± 67	82 ± 28	1.76
Protein kinase C-binding protein NELL2	NELL2	Q99435	214 ± 128	121 ± 47	1.77
Ephrin type-A receptor 1	EPHA1	P21709	229 ± 90	130 ± 48	1.76
Kallikrein-7	KLK7	P49862	170 ± 75	97 ± 42	1.75
Layilin	LAYN	Q6UX15	145 ± 78	83 ± 68	1.75
WAP four-disulfide core domain protein 2	WFDC2	Q14508	375 ± 116	217 ± 45	1.73
Fibroblast growth factor receptor 2	FGFR2	P21802	275 ± 92	163 ± 29	1.69
Somatotropin	GH1	P01241	96 ± 50	59 ± 11	1.63
Complement C5/C5a	C5/C5a	P01031	89 ± 69	55 ± 50	1.61
Zinc-alpha-2-glycoprotein	AZGP1	P25311	675 ± 144	420 ± 104	1.61
Chorionic somatomammotropin hormone 1	CSH1	P0DML2	109 ± 59	68 ± 58	1.61
Serotonin	HTR3B	O95264	221 ± 26	139 ± 58	1.59
Protein AMBP	AMBP	P02760	2890 ± 572	1830 ± 575	1.58
Tyrosine-protein kinase BTK	BTK	Q06187	616 ± 178	406 ± 128	1.52
Tyrosine-protein kinase ZAP-70	ZAP70	P43403	187 ± 23	283 ± 32	0.66
Alkaline phosphatase, placental type	ALPP	P05187	58 ± 77	93 ± 73	0.63
Somatostatin receptor type 2	SSTR2	P30874	156 ± 42	248 ± 32	0.63
Kallistatin	SERPINA4	P29622	92 ± 53	149 ± 60	0.62
E3 ubiquitin-protein ligase Topors	TOPORS	Q9NS56	140 ± 57	226 ± 54	0.62
Furin	FURIN	P09958	90 ± 78	150 ± 66	0.60
Serpin A12	SERPINA12	Q8IW75	270 ± 65	473 ± 65	0.57
E3 ubiquitin-protein ligase XIAP	XIAP	P98170	56 ± 9	102 ± 14	0.55
Lymphocyte activation gene 3 protein	LAG3	P18627	69 ± 65	144 ± 28	0.48
Platelet glycoprotein 4	CD36	P16671	67 ± 75	139 ± 83	0.48
Keratin, type II cytoskeletal 4 /Cytokeratin pep 4	KRT4	B4DRS2	954 ± 196	577 ± 581	1.65
Mitogen-activated protein kinase 8	MAPK8	A1L4K2	271 ± 102	471 ± 186	0.57
Protein kinase C alpha type	PRKCA	P17252	190 ± 78	241 ± 26	0.79
Protein kinase C gamma type	PRKCG	P05129	245 ± 127	309 ± 73	0.79
Protein kinase C beta type	PRKCB	P05771	89 ± 98	119 ± 75	0.75
DNA damage-inducible transcript 3 protein	DDIT3	P35638	61 ± 89	104 ± 62	0.59

^*^MFI: Median Fluorescence Intensity. SD: standard deviation. ^^^*p* < 0.05

Interestingly, among the deregulated proteins associated to Alzheimer’s disease, we found previously reported AD-associated proteins, including the accumulation of GATA4 and Chromogranin-A [24–27], or MBL2 [28], whose over-expression has been described in the surrounding of blood vessels in AD brain patients. In addition, we observed in our analyses the down-regulation of PKC, which has been suggested as a crucial step of AD pathogenesis [29, 30]. Collectively, our data are in agreement with scientific data regarding the over-expression or down-regulation of some of the here AD-associated identified proteins [24–31], and thus validating the identified altered deregulated protein dataset in AD.

### Biological function, pathway analysis, and interaction networks of deregulated proteins

To gain further insight into altered AD-specific pathways, interactors and clusters of proteins, subsequent bioinformatics analyses were performed to explore the identified deregulated protein dataset with Ingenuity Pathway Analysis (IPA) and STRING [14, 32]. We included in the dataset APP and Tau because of their pivotal role in AD and to analyze for potential relationships between the deregulated proteins and the neuropathological substrates.

**Figure 3:**
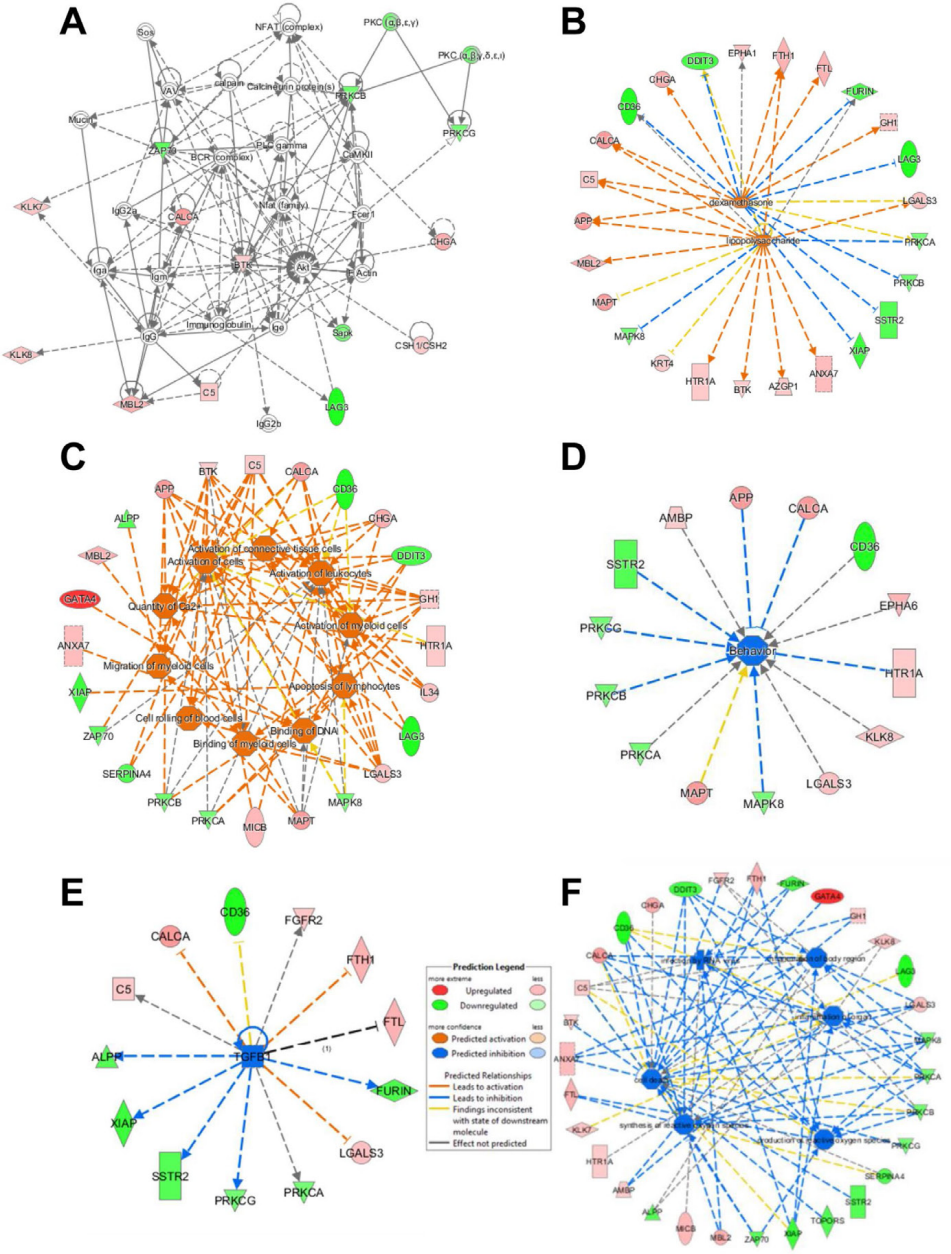
Ingenuity pathway analysis of the antibody microarray data Affected protein networks were identified using the 40 differentially expressed proteins together with APP and TAU (MAPT) identified in the study by IPA. (**A–F**) Up-regulated proteins are shown in red, and down-regulated proteins in green. (A) Cell signaling and cellular function and maintenance was the most affected network. (B) The identified proteins were found to produce an over-activation of networks involving lipopolysaccharides (LPS) and dexamethasone. (C) Among the most affected cellular functions, cell activation and the calcium amount were predicted to be activated, together with alterations in behavior (D). (E) A lesser activation of TGFβ-mediated networks was predicted, and (F) the production of reactive oxygen species was predicted to be inhibited, when APP and Tau were removed from the dataset for the analysis. Fx, function; CP, cell process.

First, differentially expressed proteins in AD tissue samples were analyzed using IPA to identify altered molecular, cellular and biological functions and pathways. Cell signaling and Cellular Function and Maintenance (15 molecules out of the 40 deregulated proteins, score 26) together with cell death and survival (11 molecules, score 21) stand out as the most altered cellular pathways in the protein dataset associated to AD deregulation (Figure 3A and Supplementary Figure 2) [33].

In addition, we analyzed which pathways would be predicted to be activated or inhibited by IPA according to our dataset. Based on the here identified up- and down-regulated proteins, the activation of lipopolysaccharide (LPS) (17 molecules, *p* value 9.92E-9) and dexamethasone (17 molecules, *p* value 1.12E-8) mediated pathways were predicted, both of which have been described to produce AD-like characteristics in animal models (Figure 3B) [34–36]. The analysis also revealed, as the most altered functions, amongst others, those related to activation of cells and the amount of calcium (19 and 9 molecules, respectively, *p* value ≤ 8.33E–7), which have been previously associated to AD (Figure 3C) [37], and alterations in behavior (14 molecules, *p* value = 7.09E–07), which is a common feature of AD patients (Figure 3D). Finally, by removing from the dataset APP and Tau, we only observed changes in those pathways predicted to be inhibited. It was observed the inhibition of the TFG-β mediated pathway (13 molecules, *p* value 2.72E-6), which has been reported to cause age-dependent neurodegeneration, Aβ accumulation, and dendrite loss in animal models (Figure 3E) [38], and alterations in the synthesis and production of reactive oxygen species (16 and 13 molecules, respectively, *p* value ≤ 5.2E–12) (Figure 3D), whose alterations have been previously related to AD (Figure 3F) [37, 39].

**Figure 4:**
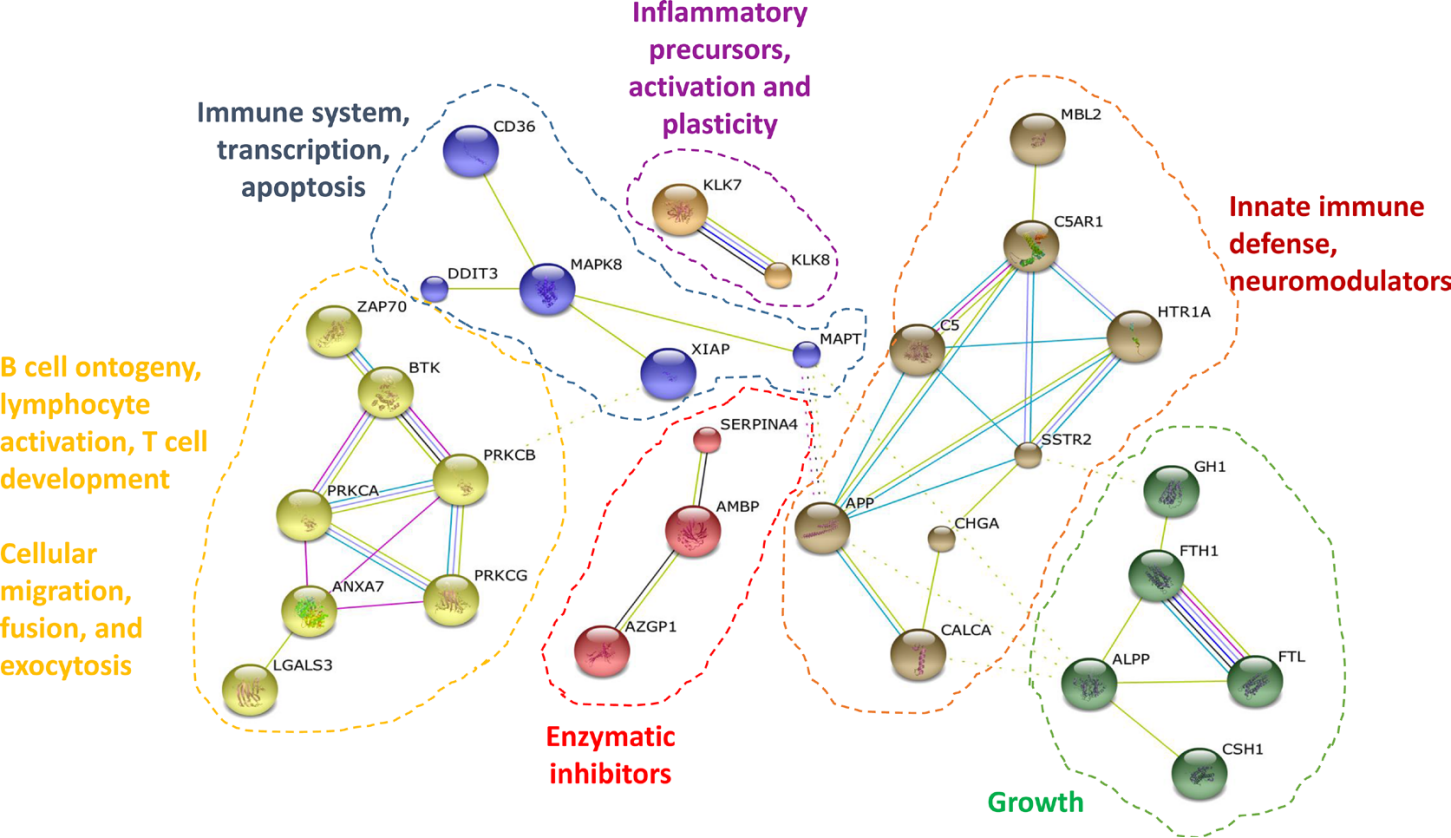
Protein interactome map of the differentially altered proteins in Alzheimer’s disease STRING analysis of known and predicted protein-protein interactions of up-regulated and down-regulated gene-products differentially expressed in AD in comparison to healthy individuals and FTD and VD patients. STRING Version 9.1 and STRING-db were used [32]. Only those clusters of 2 or more interconnected proteins are shown. Individual nodes were removed from the figure.

We then used STRING to define the deregulated interactome associated to AD using the 40 differentially expressed protein dataset together with APP and Tau [32]. Using the MCL algorithm, we observed six clusters of interactor proteins containing two or more proteins (Figure 4). It was observed that four clusters were related to the immune system, highlighting again the strong association of the deregulated Alzheimer’s disease dataset to the immune system [22, 23].

The smallest clusters of proteins related to the immune system involved DDIT3, XIAP and CD36 around MAPK8 and Tau (MAPT), which was also related to RNA transcription and apoptosis, and KLK7 and KLK8, which are involved in the activation of inflammatory precursors and neuronal plasticity [40]. On the other hand, the largest clusters of proteins composed of five and seven proteins were related to the innate immune defense and growth, and lymphocyte activation, T cell development, and B cell ontogeny, respectively. The largest clusters were involved in i) lymphocyte activation, T cell development, and B cell ontogeny, included calcium activated proteins (such as PKCs), and proteins involved in cellular migration, exocytosis and cellular fusion (such as LGALS3BP or BTK), and ii) innate immune defense around APP, which contained SSTR2 and HTR1A receptors involved in neurotransmission, neuromodulators (including Chromogranin-A and calcitonin), and MBL2, where the mannan-binding lectin pathway has been reported to trigger the complement cascade, including C5/C5AR1 [41, 42]. Finally, we also found two clusters composed of two, and five proteins, which included cellular growth proteins (such as FTL and ALPP) and enzymatic inhibitors (AMBP, SERPINA4 and AZGP1), respectively.

In summary, we observed an important number of proteins related to the immune system, cell adhesion proteins and receptors, with some of them not previously identified as altered at protein level in AD like LGALS3BP, DDIT3, AZGP1, MICB, TOPORS, Layilin or CD36. An imbalance on pathways related to cell proliferation, growth and survival identified as altered by IPA has already been reported to be associated to Alzheimer’s disease as a consequence of the neuronal cell death [33–39, 43]; and thus, verifying the association of the dataset to AD. Therefore, the knowledge of the here identified altered proteins in AD could be useful to advance in the knowledge of the disease, and may have implications in the diagnosis and management of AD patients, since some deregulated proteins could be associated to AD progression.

### Verification of the specific alterations in the prefrontal cortex of Alzheimer’s disease patients

First, we performed a meta-analysis of the mRNA expression using existing published data suitable to provide information about the correlation between transcript and protein levels of our protein dataset. To that end, we focused our attention on large *post mortem* studies examining the mRNA expression levels in the prefrontal cortex tissue from late-onset AD and control patients deposited on public databases [44, 45].

**Figure 5:**
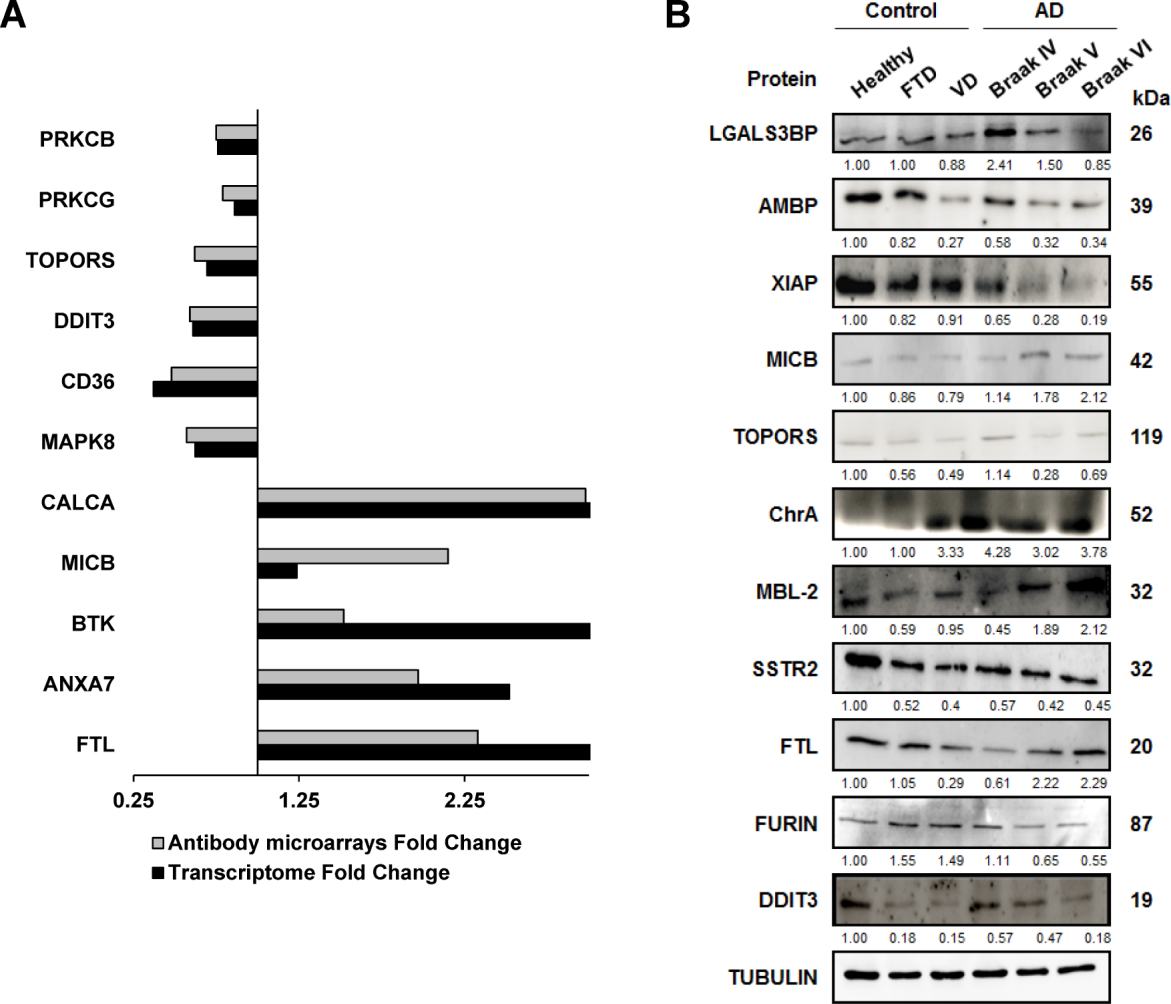
Meta-analysis of mRNA alterations and validation of protein alterations in tissue samples (**A**) mRNA alterations in the prefrontal cortex of indicated genes showed concordant significant results between results from large transcriptome studies and here reported alterations at protein level by using antibody microarrays. Transcriptome fold changes (AD/Control mRNA levels) were obtained from the meta-analysis of large transcriptome studies [44, 45, 98], and here reported antibody microarrays fold change (AD/Control protein levels, Table 2) are depicted in the figure. (**B**) A panel of antibodies against the indicated altered proteins was tested by WB analysis for verification of protein alterations using optimized dilutions of the antibodies (Supplementary Table 2). Tubulin was used as loading control in the same gels. SDS-PAGE 10% gels were run with protein extracts from the indicated group conditions and transferred onto nitrocellulose membranes. Then, membranes were incubated with specific antibodies at optimized dilutions and the signal developed with HRP-labeled secondary antibodies (Supplementary Table 2).

From the total of the 40 deregulated proteins identified in our study, IL34, XIAP, C5/C5a, and serotonin were not found in the datasets. Using the same cut-off than in the antibody microarrays, twenty-two genes were observed to be unaffected and 11 mRNAs showed concordant results with the proteomic dataset (Figure 5A). We also observed 3 genes showing opposite trends (NELL2, KLK7, and BTK), suggesting a post-transcriptional or post-translational regulation of these mRNAs or proteins, respectively. This regulation should produce the alteration in the expression of these proteins in AD, since KLK7 and NELL2 have been previously reported to be deregulated at protein level in AD [16, 40, 46], as observed in the antibody microarray analysis. In addition, we next focused our attention in determining if these alterations might also be found on other neurodegenerative conditions independently on the analyzed brain region. After extensive meta-analysis, we only found alterations at mRNA level for AZGBP1 and ALPP in Parkinson’s disease [47], and LGALS3BP in Lewy body dementia [48]; and thus, suggesting that the deregulated proteins identified in our study were mostly specific of AD.

We then analyzed changes at protein level by WB of selected proteins from those clusters containing more than two interacting proteins identified by STRING, together with interesting proteins out of the clusters selected by IPA (Figure 5B). We tested antibodies against eleven proteins (LGALS3BP, AMBP, XIAP, MICB, TOPORS, Chromogranin-A, MBL2, SSTR2, FTL, FURIN, and DDIT3) using tubulin as loading control and six different pools of protein extracts corresponding to healthy individuals, DFT, VD and three AD Braak stages (IV, V, and VI).

Remarkably, we verified the up-regulation or down-regulation for 10 out of the 11 proteins analyzed by WB (Figure 5B). We found that XIAP, TOPORS, SSTR2, FURIN and DDIT3 showed down-regulation, and LGALS3BP, MICB, Chromogranin-A, MBL2 and FTL a clear up-regulation, at similar extents than that observed in the antibody microarrays. Interestingly, we also found that the expression of XIAP, FURIN, LGALS3BP and DDIT3 decreased, whereas MICB, MBL2 and FTL expression increased in parallel to the progression of the disease. Therefore, these proteins could be potential mediators related to the progression of the disease.

Finally, we compared the identified altered proteins with those derived from mass-spectrometry based quantitative proteomics studies to determine the complementarity of both proteomics techniques for the identification of protein alterations in AD, and as another strategy to verify the data at protein level (Table 3). We only observed 6 out of the 40 altered proteins from the dataset appearing in different mass-spectrometry based AD quantitative proteomic studies [49–54], showing the complementarity of both proteomic techniques for the discovery of altered proteins in AD. In addition, among the 40 altered proteins, we only observed the accumulation of FTL in VD patients [55], after analyzing large datasets from quantitative proteomics studies related to other neurodegenerative conditions, suggesting -in addition to the meta-analysis of mRNA alterations- the specificity of the identified deregulated proteins for Alzheimer’s disease. On the other hand, it was observed a good concordance between antibody microarrays and mass-spectrometry data regarding to the alterations in the expression of 5 (FTL, LGALS3BP, MBL2, PRKCA, and PRKCG) of the 6 proteins identified using different quantitative mass-spectrometry approaches and samples from cortical areas, temporal neocortex, and hippocampus from AD patients and controls (Table 3) [49–54]. In addition, we observed non-concordant results for ANXA7 [49]. Interestingly, this proteomics study was performed using olfactory bulb samples, suggesting a potential differential deregulation of this protein in different brain areas according to the progression of AD.

**Table 3:** Altered prefrontal cortex proteins in Alzheimer’s disease identified by antibody microarrays observed in Alzheimer’s disease mass-spectrometry based proteomic studies

MS-based proteomic analysis of AD							
Protein name	Gene name	Uniprot ID	AD/Control ratio	Proteomics technique	Brain region samples	Reference	AD/Control ratio (Antibody microarrays)
Annexin A7	ANXA7	P20073	0.76	Label free	Olfactory bulb	[49]	1.97
Ferritin Light Chain	FTL	P02792	2.82	Label free	Cortical samples	[50]	2.33
			2.3	Dimethyl-Labeling	Temporal neocortex	[51]	
			2.65	iTRAQ	Frontal cortex	[52]	
			4.13	Label free	Hippocampus	[53]	
			2.21^*^	iTRAQ	BA21 area of the temporal lobe	[55]	
Galectin-3-binding protein	LGALS3BP	P17931	1.77	iTRAQ	Frontal cortex	[52]	1.86
Mannose-binding protein C	MBL2	P11226	1.85	Label free	Cortical samples	[50]	2.36
Protein kinase C alpha type	PRKCA	P17252	0.49	Label free	Hippocampus	[54]	0.79
Protein kinase C gamma type	PRKCG	P05129	0.38	Label free	Cortical samples	[50]	0.79
			0.49	Label free	Hippocampus	[54]	

^*^Fold changes = 2.21 were observed for Ferritin Light Chain (P02792) in Vascular Dementia patients analyzing the BA21 area of the temporal lobe by iTRAQ [55].

Although divergences were observed in the concordance of mRNA and protein expression levels between meta-analysis of mRNA alterations, antibody microarrays, mass-spectrometry studies and WB analyses, collectively, our results and previous scientific data validate the altered proteins identified by antibody microarray screening. The divergences could be associated to post-transcriptional and post-translational regulation producing a deregulation of the proteins in AD, to a potential bias in the pooling strategy, which may be associated to unpredictable confounders such as environmental, behavioral and agonal factors among the different groups (i.e medication, substance abuse and health status prior to death) [56], or to some artifacts in the microarrays processing and analysis [57–59]. In addition, the comparison of the antibody microarray and mass-spectrometry based studies data confirmed antibody microarrays-driven proteomics as a good approach for the identification of protein alterations in molecules implicated in cell-cell communication and cell signaling processes, and the complementarity of both proteomic approaches for the identification of protein alterations in AD.

### Immunohistochemistry and fluorescence *in situ* hybridization analyses using an Alzheimer’s disease-specific tissue microarray

Despite inter-individual variability is generally absent by analyzing pool samples, individual sample analysis with a higher number of samples is needed to validate the results. Therefore, to verify the results at protein level, we analyzed alterations in protein abundance of indicated proteins by IHC and through its potential relation with aberrations in DDIT3 gene by fluorescence *in situ* hybridization (FISH) using an AD-specific TMA. The TMA was assembled with brain tissue from the prefrontal cortex of 44 different AD patients at different Braak stages, FTD and VD patients and healthy individuals (Supplementary Table 1).

**Figure 6:**
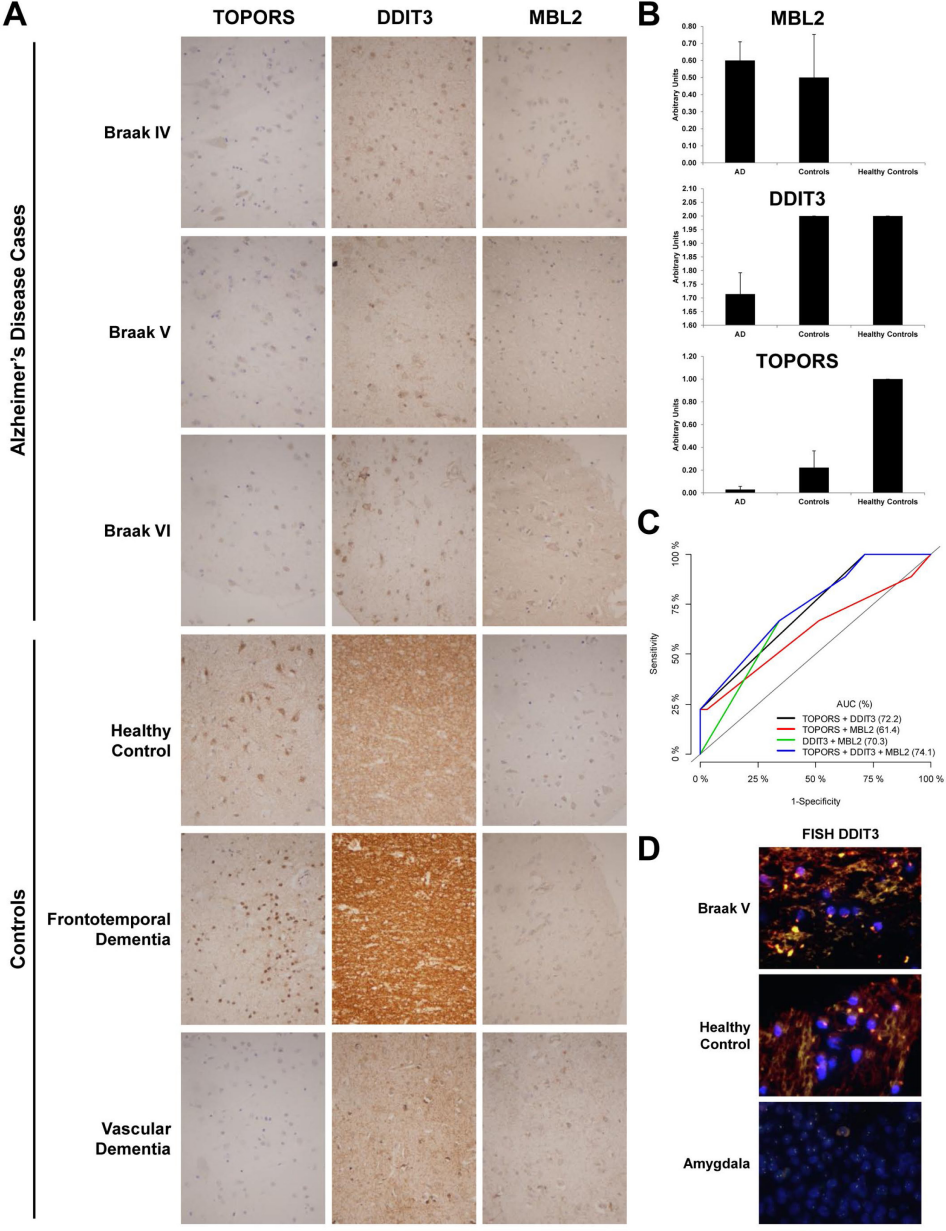
Immunohistochemical and FISH analysis of selected targets in human AD and controls (**A**) Representative immunohistochemical staining pattern of indicated proteins in AD Braak stages IV, V and VI and FTD and VD patients and healthy individuals. Depicted images shown at 200× magnification images correspond to the immunohistochemical staining of indicated deregulated proteins in AD showing discriminatory capacity between AD and control tissues using an AD-specific TMA containing 44 samples. (**B**) Quantification of the TMA depicted as bar graphs showed significant alterations for TOPORS (*p* value = 0.02), and DDIT3 (*p* value = 0.035). Although MBL2 alterations were not significant due to the low number of healthy individuals in comparison to AD group, the bar graph is depicted in the figure as control of reactivity for its already known accumulation on AD [28, 87]. VD and FTD patients and healthy individuals were represented in the bar graph as controls. Healthy controls bar represented the IHC staining only for healthy individuals in arbitrary units. (**C**) Validation of TOPORS, DDIT3 and MBL2 as AD markers by means of ROC curve analyses using different combinations of the three proteins. The AUC (%) for the different combinations to discriminate AD from controls is shown in the figure. (**D**) Representative images of cases (AD Braak V) and controls (healthy control) showing no translocations involving DDIT3 were visualized by interphase FISH in the TMA using SureFISH DDIT3 dual color break-apart for DDIT. A positive control (amygdala) is included in the figure. A positive and a negative control slide were included in the assay for the validation of the results.

We set up conditions by IHC for six proteins (MBL2, TOPORS, XIAP, DDIT3, ANXA7, and MICB) using control slides. However, in the Alzheimer’s disease specific TMA, we only obtained significant data for TOPORS and DDIT3. We also observed, as control of the performance of the TMA, the accumulation of MBL2 in AD patients in comparison to healthy individuals, as expected according to previous data [28]. Data analysis of the immunohistochemistry analyses of the TMA revealed significant data (*p* value < 0.035) for the altered expression of TOPORS and DDIT3 in AD prefrontal cortex tissue in comparison to controls (Figure 6A and 6B). The overall staining of TOPORS showed a slight down-regulation in comparison to healthy individuals. However, the down-regulation of TOPORS became highly significant at glia, comparing AD and healthy individuals (Figure 6A and 6B). In addition, we also observed TOPORS down-regulation in VD and FTD, suggesting that TOPORS down-regulation is a common event in neurodegeneration. The other significant alteration in protein expression was related to the down-regulation of DDIT3, which was specific of AD patients in comparison to healthy individuals or VD and FTD patients (Figure 6A and 6B). Then, with these data, we generated ROC curves to evaluate the individual performance of DDIT3, TOPORS and MBL2 and their different combinations as AD markers (Figure 6C). Although the individual AUC of DDIT3, TOPORS, and MBL2 was 64.3%, 59.7% and 57.6%, respectively, the combination of TOPORS and DDIT3 reached an AUC of 72.2%, and the combination of TOPORS, DDIT3 and MBL2 increased the AUC to 74.1% with a sensitivity of 66.7% and a specificity of 66.7%, indicating the good performance of these proteins in discriminating AD samples from controls.

Finally, since several recent reports have observed by FISH the presence of large-scale genomic alterations in neural cells of AD patients related to AD pathogenesis (i.e. aneuploidy, X-chromosome instability, APP duplications…) [60–62], we decided to explore the potential existence of rearrangements in the DDIT3 gene, which would be responsible for the down-regulation of DDIT3 at mRNA and protein levels in AD (Figure 5). However, we did not perceive any alteration in the DDIT3 gene (Figure 6D), suggesting that DDIT3 alterations at mRNA and protein level should be associated to post-translational modifications.

Collectively, a consistent trend was observed among the proteomic results derived from the antibody microarrays and the validation results at protein level obtained by WB and IHC. Indeed, in accordance with our proteomic findings, we observed an interesting deregulation of the E3 ubiquitin ligases (TOPORS and XIAP), which together with the non-previously related to AD deregulation at protein level of LGALS3BP, Layilin, MICB, CD36 and DDIT3 constitute interesting proteins for further functional analysis to determine their role in AD.

## DISCUSSION

Encouraged by the idea of monitoring signaling-associated proteins in AD, which are fundamental in cell-cell communication and cell signaling processes and frequently missed in mass spectrometry-based proteomics, we performed antibody microarrays-driven proteomics analysis of *post mortem* tissue from AD patients and controls. We used antibody microarrays to specifically monitor alterations in 706 proteins biased to immunomodulators, cytokines, chemokines, adipocytokines, growth factors, and proteases, motivated by the idea that after further studies they might become potential blood-based biomarkers of the disease, as most of these molecules are secreted.

Most of the proteomics-based studies focused on the analysis of Alzheimer’s Disease are related to the comparison of healthy individuals *vs* AD cases (*for reviews see* [4, 5, 9, 10]), by analyzing different brain areas affected by the disease [63–66], or mainly CSF, serum or plasma [5, 9, 11]. Then, the obtained data are verified using higher cohorts of samples, where patients with other dementias are then included. Therefore, results might be potentially biased to the identification of a common fingerprinting of neurodegeneration. Here, to overcome this problem, and minimize any bias in the pooling strategy due to a low number of samples in the healthy and vascular dementia group, we performed the discovery of AD protein alterations by comparing control samples with AD samples. We used tissue from the prefrontal cortex from healthy individuals and frontotemporal dementia, and vascular dementia patients (control group), and AD samples from patients in Braak stages IV, V and VI (AD group) to identify AD-specific alterations non-related to other dementias.

Antibody microarrays enabled the discovery of forty differentially expressed proteins in neuropathological confirmed AD patients compared to healthy individuals and VD and FTD subjects (Table 1). Remarkably, previous and here reported data pointed out to the validity of the dataset, including: i) the identification of previously reported altered proteins in AD, like the upregulation of GATA4, Chromogranin-A, or MBL2 and the downregulation of PKC [24–31], ii) the deregulation of pathways previously associated to AD, like calcium homeostasis, TFGβ mediated pathway, or the downregulation of cellular proliferation and growth and the production of reactive oxygen species [33, 37–39], iii) the meta-analysis of mRNA alterations showed concordant results with the protein alterations identified by antibody microarrays for 11 mRNAs, iv) the concordance in the expression levels of 5 altered proteins also observed in mass-spectrometry based AD proteomic studies, and v) the validation of 10 of the deregulated proteins by WB and IHC. In addition, we were able to confirm the complementarity of antibody microarrays and mass-spectrometry proteomics techniques for the identification of protein alterations in Alzheimer’s disease, since we only found 6 out of the 40 altered proteins identified by antibody microarrays in reported datasets from mass-spectrometry studies [49–54].

One of the most interesting findings of the study consisted of the identification of the alteration of two E3 ubiquitin-protein ligases: XIAP and Topors, which has not been previously associated to AD, apart from the relevant association of 15 proteins and four of the six clusters of interacting proteins to the immune system, which highlight the important role of the inflammatory response in the AD pathogenesis [22, 23]. We observed a clear downregulation of these ubiquitin ligases in AD cases in comparison to controls, which was further confirmed by WB and IHC. XIAP expression in AD remains controversial [67, 68], and Topors deregulation or its relationship with AD unexplored. Our results related to the down-regulation of XIAP are in agreement with the observed downregulation of PKC in our dataset and in previous works related to AD [31, 69], where PKC stabilizes XIAP through phosphorylation to suppress apoptotic cell death [31] and its cross-talk with XIAP has been suggested to be crucial in regulating the impaired neuronal homeostasis [31]. XIAP is an anti-apoptotic protein that binds to caspases 3, 7, and 9, inhibiting their action [70]. Therefore, a decrease on XIAP and Topors would produce an unbalanced apoptosis regulation that should contribute to the neuronal death observed in the brain of AD and other dementia patients. Furthermore, different studies have shown a decrease in the levels of other ubiquitin ligases involved in Aβ ubiquitination and protein clearance in AD patients, such as Parkin [71]. Beyond E2 conjugating enzymes, E3 ligases, and de-ubiquitinating enzymes have been shown to play a pivotal role in the proteasomal degradation of Aβ [72–74]. Remarkably, XIAP downregulation was observed to be specific of AD, whereas Topors expression seemed to be a common event present in AD, VD and FTD. Further functional analysis focused on the mechanism of these ubiquitin ligases in AD and neurodegeneration may provide alternative therapeutic targets and lead to new drugs and therapies.

One of the goals of the study was to generate validated data on the here identified deregulated proteins involved in the pathology. Although sample pooling strategy reduces false-positive rates in proteomics mitigating for individual clinical and pathophysiological heterogeneity, we verified the alteration of proteins belonging to interesting clusters of interaction selected by bioinformatics analyses using individual samples. Although we included in the antibody microarray analysis healthy controls as well as patients with other dementias, to determine to what extent the observed protein alterations were specific of AD or neurodegeneration, we also analyzed individual samples from 44 AD patients, and healthy individuals, VD and FTD patients as controls by WB and IHC using an AD-specific TMA. Apart from the analyzed ubiquitin ligases, we surveyed for the expression of proteins non-previously associated to the disease like LGALS3BP, layilin, and MICB; and thus, constituting interesting targets for further analysis to get further insights into their association to the disease.

Therefore, we analyzed the tissue protein expression of LGALS3BP, with no clear validated data about its expression levels in AD patients [52, 75–77]. LGALS3BP is a glycoprotein that promotes integrin-mediated cell adhesion and may stimulate host defense against viruses and tumor cells. It is implicated in immune response by its association with natural killer and lymphokine-activated killer cell cytotoxicity [78]. Its production can be triggered through LPS, which has been found to cause AD-like symptoms in animal models [34, 35, 79]. Here, although we observed a global upregulation of the protein, its expression decreased with the progression of the disease, whose implications in AD should be further investigated.

An increase for layilin and MICB proteins was also found. Layilin, a hyaluronan receptor, is suggested to play a role in cell adhesion and motility by mediating early interaction between cells and the extracellular matrix [80]. Its observed increased protein levels would imply a deregulation on adhesion and motility in AD. MICB is a glycosylated stress-induced protein that activates, amongst other immune responses, natural killer cells [81]. Even though there is not known any relationship between this molecule and AD, it can be speculated that the protein may be secreted due to the stressful environment that the AD brain cells are, and thus contributing to enhance the immunological response observed in the disease. Therefore, layilin and MICB constitute interesting targets to determine their role in the disease in subsequent analysis.

We also focused our attention on two controversial proteins in AD: DDIT3 and MBL2. Previous studies have shown an increase, absence or repression of DDIT3 in AD patients [82]. DDIT3 acts with NFκ-β to regulate β-secretase (BACE1) expression, which cleaves APP, leading to aberrant Aβ production [83]. A decrease on its levels could be related to the over-activation of BACE1 and, consequently, a higher Aβ production. We found a decrease on the protein levels for DDIT3 in AD, and thus, it would constitute an interesting target to determine the exact role of the protein in the disease. On the other hand, MBL2 activates the complement system and binds to apoptotic and senescent proteins to facilitate their phagocytosis. Therefore, an increase on its levels could contribute to the neuronal death observed in AD [84–86]. However, controversial data exists about its role in the disease. Previous studies have shown no difference in its brain distribution in patients with AD in comparison to control groups, and a decrease on its levels in AD CSF [28, 87]. The fact that these studies found an increase on protein levels in the tissue samples does not contradict the data found in CSF [28, 50, 87]. It could mean that MBL2 expression is higher in AD or that it is clearance through the CSF is impaired, and thus heightening both its presence and activity in the brain.

Finally, we have also here demonstrated by immunohistochemistry the good performance of two of these proteins –DDIT3 and Topors- in combination to MBL2 for the discrimination between AD cases and controls by means of ROC curve analyses. These proteins showed in combination a good ability to identify AD samples from controls with an AUC of 74.1% with a sensitivity of 66.7% and a specificity of 66.7%, which demonstrated the usefulness of the followed approach to identify altered proteins as novel markers in AD.

A similar antibody microarray approach was recently tried for the screening of 584 signaling proteins in plasma samples [16]. Coincident data among the reported data in plasma [16], and the here observed protein alterations in brain tissue were observed like the deregulation of TGFβ-mediated networks, and the upregulation of FTL and the downregulation of FURIN in plasma of AD patients [16]. Remarkably, the here presented approach was thought as a first step for a further elucidation of selected targets as blood-based biomarkers after extensive immunological analysis of sera or plasma of AD patients by ELISA or as target of intervention after functional analysis. Coincident results among both antibody microarray studies suggest that some of the protein alterations observed in the brain tissue of AD patients could also be observed as molecular changes in the blood or CSF of AD patients. In addition, it has also been recently showed in a systematic review that 18 out of 371 differentially expressed proteins identified by proteomics in the brain of AD patients were also present in blood proteomic studies of AD [88]. Therefore, these coincident results support the initial idea of the study, encouraging us to perform subsequent immunological analyses to detect their presence in sera, plasma or CSF to determine the usefulness of the altered signaling molecules LGALS3BP, Layilin, MICB, and DDIT3 as blood- or CSF-based AD diagnostic biomarkers. Furthermore, our results also encouraged us to perform functional analysis focused on the mechanism of action of interesting deregulated targets like the E3 ubiquitin ligases XIAP and TOPORS or CD36 in AD, which could become alternative targets of intervention in the disease.

## CONCLUSIONS

We have identified 40 altered proteins in AD brain tissue using an antibody microarrays-based proteomics approach for the probe-directed analysis of molecules implicated in cell-cell communication and cell signaling processes. We used as controls other forms of dementia and healthy individuals to obtain AD-specific altered proteins non-related to other dementias. The differential deregulated proteome lies in an imbalance in several immunological processes, emphasizing its role in the disease, together with the deregulation of pathways previously associated to AD, like calcium homeostasis, TFGβ-mediated pathway, or the downregulation of cellular proliferation and growth and the production of reactive oxygen species. Our findings provide validated novel altered proteins -LGALS3BP, Layilin, MICB, CD36, DDIT3, TOPORS and XIAP- non-previously associated to AD that should be further explored in subsequent studies to determine their usefulness as blood or CSF biomarkers and should be the focus of functional experiments to determine their role in AD to potentially identify new targets of AD intervention.

## MATERIALS AND METHODS

### Samples

Tissue samples with indicated pathological conditions were obtained from the CIEN Foundation’s Tissue Bank (BT-CIEN). According to the brain bank’s protocols, neuropathological diagnosis and classification of cases was performed on the basis of international consensus criteria [1, 89, 90]. The BT-CIEN develops a brain donation program based on SOPs, meeting the ethical and legal requirements established by current legislation regarding the protection of personal data procedures and as regards to the use of samples of human origin for biomedical research. Written informed consent was obtained from all patients. The Institutional Ethical Review Board of the Spanish Research Center for Neurological Diseases Foundation (CIEN) and the Complutense University of Madrid approved this study on proteomic analysis of Alzheimer’s disease.

A total of 44 brain tissue samples from the left prefrontal cortex of cases and controls were used in the study (Supplementary Table 1). Thirty-five tissue samples from sporadic AD patients’ brains ranging from Braak IV to VI and a group of 2 healthy individuals, 5 patients with Frontotemporal Dementia (FTD) and 2 patients with Vascular Dementia (VD) were used as controls. The average age and standard deviation of all samples was 80.84 ± 9.90 years (range 55–98 years).

### Protein extraction

Protein extraction was performed as previously reported [91–93]. Briefly, tissue samples were cut in small pieces in dry ice and mechanically disaggregated with SDS 0.5% in phosphate buffered saline (PBS) with a protease inhibition cocktail (Sigma), and finally clarified by centrifugation at 10000 rpm.

Protein soluble extracts quality was assessed by Coomassie staining of SDS-PAGE 10% gels [92]. Protein samples were pooled into six different groups according to their pathological characteristics (healthy subjects, FTD patients, VD patients, and AD patients ranging from Braak stages IV to VI). Protein concentration of all the extracts was determined by bicinchoninic acid assay (Thermo Scientific), before pooling to ensure equal total protein from all the individual samples.

### Antibody microarrays

We used two different antibody microarrays from two different commercial sources for the profiling of 706 proteins mostly implicated in cell-cell communication and cell signaling processes. The antibody microarrays permitted the profiling of 706 different proteins, with 11 proteins coincident in both arrays (Caspase 3, Caspase 8, Cathepsin D, Cyclin D1, Cytokeratin pep 18, Fibronectin, HSP70, HSP90, Pyk2, S-100b, and SMAC).

RayBio Label-Based (L-series) Human Antibody Arrays 493, consisting of two equal subarrays containing 493 unique antibodies, positive and negative controls spotted in duplicate, for the simultaneous detection of multiple cytokines, chemokines, adipokines, growth factors, angiogenic factors, proteases, soluble receptors, and soluble adhesion molecules, were obtained from RaybioTech. Microarrays were probed according to the manufacturer instructions using biotin-labeled samples followed by the incubation with streptavidin-Cy3.

Panorama Ab Microarray-Cell Signaling antibody microarrays were purchased from Sigma-Aldrich and used as per manufacturer recommendations using Cy3 and Cy5 labeled samples for the detection of key cellular proteins with a special emphasis on cell signaling proteins. Each slide contained 224 antibodies printed in duplicate onto a 32-grid array, each one containing seven antibody duplicates plus a Cy3- and Cy5-conjugated BSA positive control and a non-labeled BSA negative control. For normalization purposes, antibodies to housekeeping proteins (actin, myosin, and tubulin) were included in the arrays as their remained constant between physiological and pathological samples. Cy3 and Cy5 fluorescent dyes were from GE Healthcare.

### Sample labeling and antibody microarray screening

Pooled samples of three AD groups (Braak stages IV, V, and VI), and three control groups (healthy individuals, and FTD, and VD dementia) were labeled following manufacturer recommendations and probed in the antibody microarrays. The same samples were probed in both commercial antibody microarrays according to the manufacturer instructions.

For Panorama Antibody Microarrays-Cell Signaling Kit (Sigma), the simultaneous incubation of two samples (one control and one AD pathological condition) labeled with Cy3 or Cy5 was performed. Labeling of the samples with Cy3 and Cy5 was performed according to the manufacturer instructions. After labeling, protein concentration was determined as indicated above and the correct incorporation of the dyes confirmed by spectrophotometry [93]. Then, 20 μg of a Cy3-labeled sample, and 20 μg of a Cy5-labeled sample were simultaneously added to each microarray. A dye-swap was performed for two of the samples to survey for dye label-specific differences in antigen-antibody interactions. After 30 min incubation at room temperature with gentle shaking, the microarrays were washed and dried by centrifugation at 1200 rpm for 10 min and scanned at 532 nm and 635 nm.

For RayBio Label-Based (L-series) Human Antibody Arrays 493, the indicated samples labeled with biotin were separately incubated on three arrays containing two equal subarrays. For biotin labeling, 30 µg of tissue lysates were incubated during 30 min at room temperature with gentle shaking with labeling reagent solution. The reaction was stopped with 3 µl of stop solution, and then the samples were dialyzed to remove free biotin. Prior to the overnight incubation at 4° C onto the microarrays with gentle shaking of 3.3 µg of indicated biotinylated samples on each subarray on 400 µl of blocking buffer, the protein concentration was measured as above and the correct incorporation of biotin was determined by WB analysis using HRP-labeled streptavidin, as quality control of the labeling (data not shown). After washing, bound proteins were detected with the incubation of Cy3-streptavidin during 2 h at room temperature. Finally, after washing, the slides were dried by centrifugation at 1200 rpm for 10 min and scanned at 532 nm.

The slides were scanned on the GenePix 4000B (Axon) 2-laser scanner and images generated with the GenePix Pro 7.1 scanarray software [91, 93, 94].

### Bioinformatics and statistical analysis

Analysis, normalization, and quantification of all microarray images were performed using the GenepixPro 7.1 software. Each spot was defined by positioning of a grid given by the manufacturer. The dynamic range of the intensity, the signal to noise ratio for both microarrays, and the median values of the spots and background were determined, and interarray median normalization was performed [18, 91, 93, 95, 96]. Ratios ≥1.5 or ≤0.67 of AD (Braak IV, V and VI) *vs* controls (healthy individuals, FTD and VD patients) were used as cut-off to determine protein expression alterations, as previously done [19–21].

After normalization and identification of the deregulated AD-associated protein dataset, bioinformatic analysis was performed using String (http://string-db.org/) and Ingenuity Pathway Analysis (Ingenuity Systems, www.ingenuity.com) to identify altered networks and pathways [97]. STRING Version 9.1 and MCL clustering enrichment 2 with the default 0.4 confidence score were used to identify the interacting partners in the dataset.

To examine the mRNA expression levels in the prefrontal cortex tissue from late-onset AD and control patients to compare mRNA expression alterations in AD with our protein dataset obtained from the protein microarrays, we performed the meta-analysis of two large studies [98]. We used the GSE44772 dataset containing the analysis of the prefrontal cortex of 230 AD patient’s and controls [44], and the transcriptional analysis of 765 Alzheimer’s Disease and 669 control samples from the GSE1297, GSE5281, GSE15222, GSE26927, GSE29378, GSE29652, GSE36980, GSE37263 and GSE44772 datasets [45], respectively. In addition, we also examined other large datasets related to other neurodegenerative conditions to determine the specificity of the dataset for AD.

To examine the concordance between protein alterations identified by antibody microarrays and mass-spectrometry studies, we performed the meta-analysis of large quantitative proteomic studies involving the analysis of AD identified by a systematic search conducted in Pubmed including the terms “Alzheimer’s disease”, “human brain”, “proteomics” and “quantitative proteomics”. In addition, we also analyzed other proteomics studies involving other neurodegenerative conditions (Vascular Dementia, Parkinson Disease, Lewy Body, and Frontotemporal Dementia) to determine if the altered proteins were specifically associated to AD or were a common event in neurodegeneration.

ROC curves were constructed with the R program (version 3.2.3) using the ModelGood package and the maximized sensitivity and specificity values were calculated using the R package Epi [99].

### Antibodies

Antibodies used at optimized dilutions (Supplementary Table 2) were provided from the Human Protein Atlas (HPA) for validation of the data by WB and immunohistochemistry [100], or obtained from different sources for WB validation.

### Western blot analysis

15 μg of each protein sample were separated on 10% SDS-PAGE and transferred to nitrocellulose membranes. After 1 h blocking with PBS containing 0.1% Tween 20 (PBST) supplemented with 3% non-fat milk, the membranes were incubated overnight with indicated specific antibodies at optimized dilutions (Supplementary Table 2). The next day, after extensive washing with PBST, membranes were incubated for 1 h with HRP-secondary antibodies (Supplementary Table 2). The membranes were then extensively washed and bands visualized with WesternBright Quantum HRP substrate (Advansta). The abundance of the proteins in WB assays was quantified by densitometry using Quantity One 1D Analysis Software (Bio-Rad Laboratories) [101].

### Tissue microarray

Samples from 44 AD patients at Braak stages IV-VI and VD, FTD and healthy individuals as controls were used to construct a paraffin block containing 58 cores (1 core per patient plus 8 liver cores and 6 amygdala cores as controls) to allow immunohistochemistry analysis. A hollow needle was used to obtain a tissue core of 1 mm in diameter from selected tissue regions in formalin-fixed paraffin-embedded tissues (FFPE). These tissue cores were then inserted in a paraffin block resembling a tissue microarray. Sections from this FFPE TMA block were cut in a microtome and mounted on a microscope slide to be analyzed by immunohistochemistry and FISH.

### Immunohistochemistry and quantification

Immunohistochemical staining was conducted in 4 µm FFPE tumour sections. Slides were deparaffinized by incubation at 60° C. Biopsies were cut and incubated with PT-Link (Dako) for 20 min at 95° C in a high pH buffered solution. To block endogenous peroxidase, holders were incubated with peroxidase blocking reagent (Dako). Biopsies were stained for 20 min with optimized antibody dilutions (Supplementary Table 2) followed by incubation with the appropriate anti-Ig horseradish peroxidase-conjugated polymer (EnVision, Dako) to detect antigen-antibody interaction. Sections were then visualized with 3,3′-diaminobenzidine for 5 min and counterstained with haematoxylin. Immunoreactivity was graded as 0, absent; 1, mild staining; 2, moderate staining; or 3, intense staining as previously performed [94]. We classified the cases according to, both, the intensity of the staining and the percentage of areas showing reaction. In all cases, an external negative control was included.

### Fluorescence *in situ* hybridization

Translocations involving DDIT3 were analyzed by interphase FISH in a FFPE TMA using SureFISH DDIT3 dual color break-apart for DDIT. Cases with break-apart signals in ≥15% of the nuclei were considered positive for the presence of DDIT3 translocation. Positive and negative control slides were included with every batch for the validation of the assay.

## SUPPLEMENTARY MATERIALS FIGURES AND TABLES





## Abbreviations

AD: Alzheimer’s disease
FTD: Frontotemporal dementia
VD: Vascular dementia
FISH: Fluorescence *in situ* hybridization
IHC: Immunohistochemistry
TMA: Tissue microarray
CSF: Cerebrospinal fluid
MS: Mass spectrometry
SDS-PAGE: Sodium dodecyl sulfate-polyacrylamide gel electrophoresis
