# Supplementary material for: Identification of prefrontal cortex protein alterations in Alzheimer’s disease
Source: Oncotarget. 2018 Jan 24;9(13):10847–67. doi: 10.18632/oncotarget.24303 (PMC5834268; doi:10.18632/oncotarget.24303)
Supplement: Supplementary file 1 [file oncotarget-09-10847-s001.pdf]

Identification of prefrontal cortex protein alterations in Alzheimer's disease

SUPPLEMENTARY MATERIALS

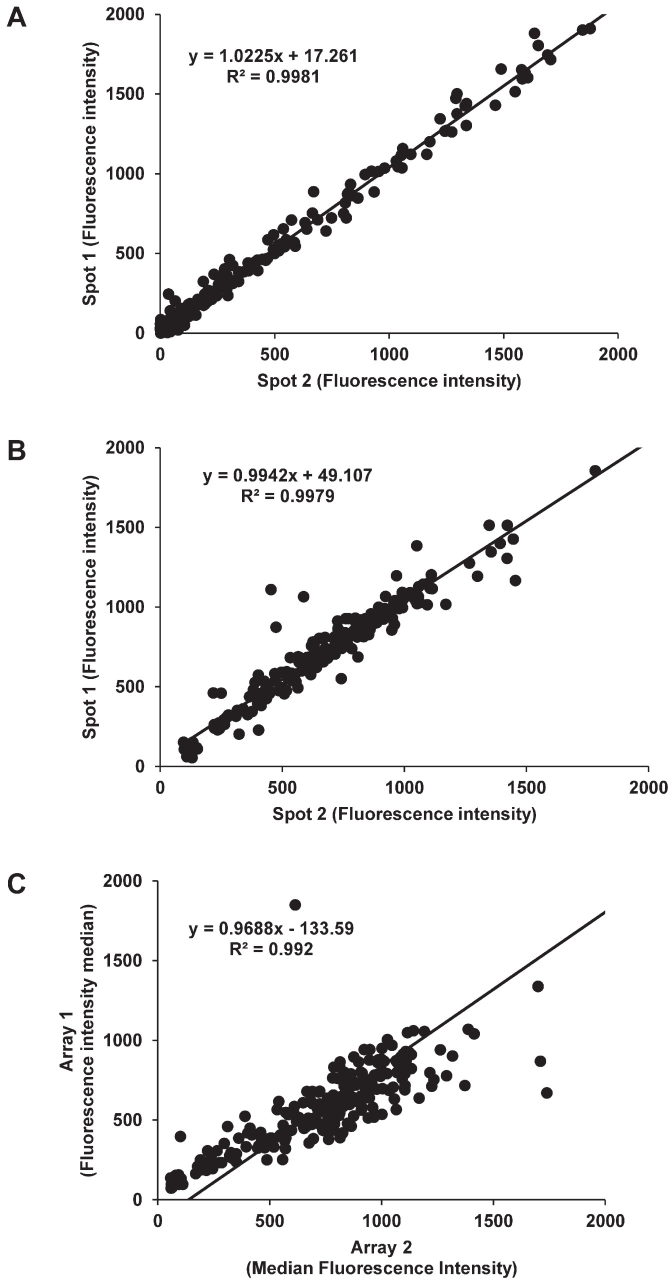

Supplementary Figure 1: Performance analysis of the microarrays. Intra-array reproducibility was determined by plotting the fluorescence intensity of duplicate spots of antibodies printed on Sigma Panorama (**A**) and RayBio L-series (**B**) antibody microarrays. (**C**) Inter-array reproducibility was calculated using the median fluorescence intensities of two different Sigma Panorama antibody microarrays probed with the swapping experiment. All samples to be tested onto RayBio L-series antibody microarrays were labeled with biotin, and then a swapping experiment was not needed.

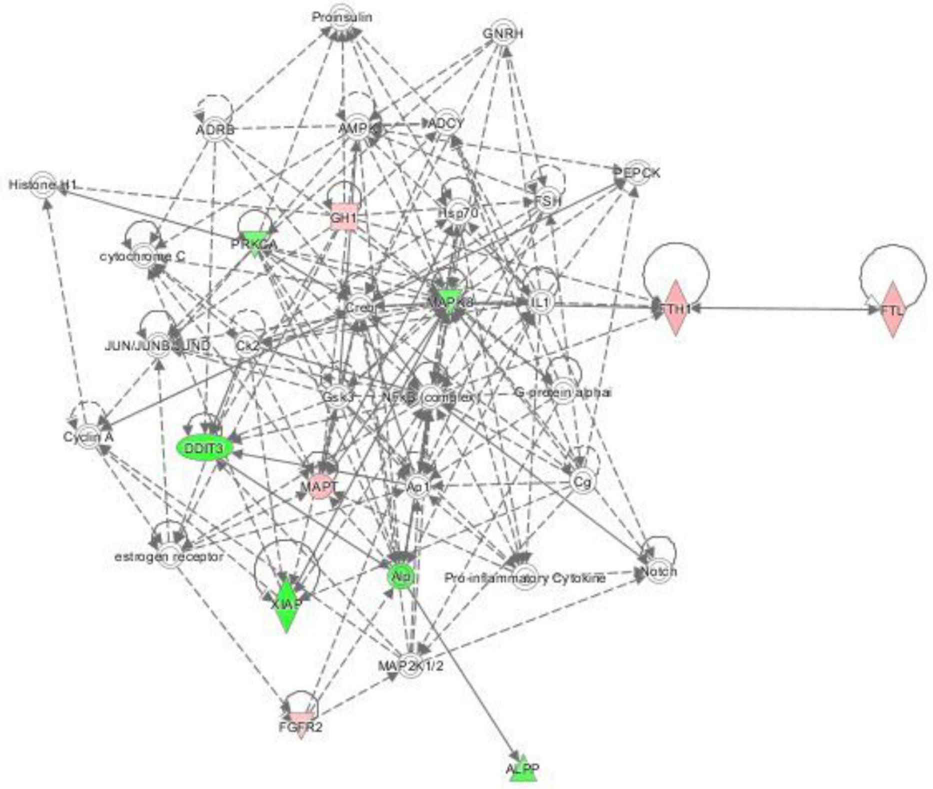

Supplementary Figure 2: Ingenuity pathway analysis of the antibody microarray data. Cell death and survival was the other most affected network identified by IPA using the 40 differentially expressed proteins identified in the study. Up-regulated proteins are shown in red, down-regulated proteins in green, uncolored proteins are proposed by IPA.

Supplementary Table 1: Sociodemographic and neuropathological data of all subjects included in the study. See Supplementary_Table_1

Supplementary Table 2: Antibodies and reagents used for the validation of the protein alteration in the study by WB and IHC

Provider	Protein	Reference	Clonality	Source	WB	IHC
Abnova	TOPORS	H00010210-M01	Monoclonal	Mouse	1:1000	1:500
Assay Biotechnology	DDIT3	C0202	Polyclonal	Rabbit	1:500	
GeneTex	AMBP	GTX101068	Polyclonal	Rabbit	1:1000	
Human Protein Atlas	SSTR-2	HPA074834	Polyclonal	Rabbit	1:500	
Human Protein Atlas	MBL2	HPA002027	Polyclonal	Rabbit	1:500	1:100
Human Protein Atlas	DDIT3	HPA068416	Polyclonal	Rabbit		1:100
Human Protein Atlas	MICB	HPA013733	Polyclonal	Rabbit	1:500	1:600
Human Protein Atlas	FURIN	HPA067869	Polyclonal	Rabbit	1:500	
Human Protein Atlas	FTL	HPA073112	Polyclonal	Rabbit	1:1000	
Human Protein Atlas	ANXA7	HPA063190	Polyclonal	Rabbit		1:50
Santa Cruz Biotechnologies	XIAP	sc-8789	Polyclonal	Goat	1:500	1:100
Santa Cruz Biotechnologies	Chromogranin-A	sc-1488	Polyclonal	Goat	1:25	
Santa Cruz Biotechnologies	LGALS3BP	sc-365338	Monoclonal	Mouse	1:1000	
Sigma	α-tubulin	T9026	Monoclonal	Mouse	1:5000	
HRP-labeled secondary antibodies or HRP-labeled reagents						
BioRad	HRP anti-rabbit		Polyclonal	Goat	1:3000	
Dako	HRP anti-mouse		Polyclonal	Goat	1:2500	
Jackson	HRP anti-goat		Polyclonal	Rabbit	1:10000	
RayBiotech	HRP-streptavidin		-		1:1000	
