# Supplementary material for: Identification of prefrontal cortex protein alterations in Alzheimer’s disease
Source: Oncotarget. 2018 Jan 24;9(13):10847–67. doi: 10.18632/oncotarget.24303 (PMC5834268; doi:10.18632/oncotarget.24303)
Supplement: Supplementary file 2 [file oncotarget-09-10847-s002.docx]

**Supplementary Table 1: Sociodemographic and neuropathological data of all subjects included in the study**

| **Group^** | **Sample Number** | **Brain Region^** | **Gender** | **Age (years)** | **Neuropathological diagnosis** | **Braak stage** |
| --- | --- | --- | --- | --- | --- | --- |
| Control | 8 | Prefrontal Cortex | M | 87 | Vascular Dementia | II |
| Alzheimer's Disease | 12 | Prefrontal Cortex | F | 74 | Alzheimer's Disease | V |
| Alzheimer's Disease | 13 | Prefrontal Cortex | F | 92 | Alzheimer's Disease | IV |
| Alzheimer's Disease | 18 | Prefrontal Cortex | F | 87 | Alzheimer's Disease | V |
| Alzheimer's Disease | 21 | Prefrontal Cortex | F | 77 | Alzheimer's Disease | VI |
| Alzheimer's Disease | 34 | Prefrontal Cortex | F | 86 | Alzheimer's Disease | V |
| Alzheimer's Disease | 41 | Prefrontal Cortex | F | 73 | Alzheimer's Disease | V |
| Control | 53 | Prefrontal Cortex | F | 93 | Vascular Dementia | II |
| Alzheimer's Disease | 57 | Prefrontal Cortex | F | 84 | Alzheimer's Disease | V |
| Alzheimer's Disease | 61 | Prefrontal Cortex | F | 83 | Alzheimer's Disease | V |
| Alzheimer's Disease | 67 | Prefrontal Cortex | F | 91 | Alzheimer's Disease | IV |
| Alzheimer's Disease | 106 | Prefrontal Cortex | F | 88 | Alzheimer's Disease | V |
| Alzheimer's Disease | 139 | Prefrontal Cortex | F | 82 | Alzheimer's Disease | V |
| Alzheimer's Disease | 153 | Prefrontal Cortex | M | 78 | Alzheimer's Disease | V |
| Alzheimer's Disease | 9 | Prefrontal Cortex | F | 98 | Alzheimer's Disease | IV |
| Alzheimer's Disease | 11 | Prefrontal Cortex | F | 88 | Alzheimer's Disease | IV |
| Alzheimer's Disease | 16 | Prefrontal Cortex | F | 77 | Alzheimer's Disease | VI |
| Alzheimer's Disease | 20 | Prefrontal Cortex | M | 71 | Alzheimer's Disease | V |
| Alzheimer's Disease | 22 | Prefrontal Cortex | M | 86 | Alzheimer's Disease | V |
| Alzheimer's Disease | 26 | Prefrontal Cortex | M | 71 | Alzheimer's Disease | VI |
| Alzheimer's Disease | 27 | Prefrontal Cortex | F | 88 | Alzheimer's Disease | VI |
| Alzheimer's Disease | 30 | Prefrontal Cortex | M | 68 | Alzheimer's Disease | VI |
| Alzheimer's Disease | 31 | Prefrontal Cortex | M | 82 | Alzheimer's Disease | V |
| Alzheimer's Disease | 32 | Prefrontal Cortex | F | 88 | Alzheimer's Disease | VI |
| Alzheimer's Disease | 36 | Prefrontal Cortex | M | 85 | Alzheimer's Disease | VI |
| Alzheimer's Disease | 37 | Prefrontal Cortex | M | 77 | Alzheimer's Disease | VI |
| Alzheimer's Disease | 39 | Prefrontal Cortex | F | 80 | Alzheimer's Disease | V |
| Alzheimer's Disease | 45 | Prefrontal Cortex | M | 66 | Alzheimer's Disease | V |
| Control | 52 | Prefrontal Cortex | F | 72 | Frontotemporal Dementia | I |
| Control | 54 | Prefrontal Cortex | F | 66 | Frontotemporal Dementia | III |
| Alzheimer's Disease | 72 | Prefrontal Cortex | M | 80 | Alzheimer's Disease | IV |
| Alzheimer's Disease | 73 | Prefrontal Cortex | F | 88 | Alzheimer's Disease | V |
| Control | 75 | Prefrontal Cortex | M | 67 | Frontotemporal Dementia | 0-I |
| Alzheimer's Disease | 78 | Prefrontal Cortex | M | 75 | Alzheimer's Disease | VI |
| Alzheimer's Disease | 110 | Prefrontal Cortex | M | 82 | Alzheimer's Disease | V |
| Control | 119 | Prefrontal Cortex | M | 55 | Frontotemporal Dementia | IV |
| Alzheimer's Disease | 123 | Prefrontal Cortex | F | 92 | Alzheimer's Disease | V |
| Alzheimer's Disease | 124 | Prefrontal Cortex | F | NA | Alzheimer's Disease | VI |
| Alzheimer's Disease | 131 | Prefrontal Cortex | F | 80 | Alzheimer's Disease | VI |
| Alzheimer's Disease | 143 | Prefrontal Cortex | M | 88 | Alzheimer's Disease | IV |
| Alzheimer's Disease | 151 | Prefrontal Cortex | F | 86 | Alzheimer's Disease | VI |
| Control | 177 | Prefrontal Cortex | F | 58 | Healthy individual | - |
| Control | 200 | Prefrontal Cortex | F | 89 | Frontotemporal Dementia | VI |
| Control | 279 | Prefrontal Cortex | F | 98 | Healthy individual | - |

^ The pathophysiological assessment was performed by immunohistochemistry at the BT-CIEN Tissue Bank of the Spanish Research Center for Neurological Diseases Foundation (CIEN Foundation) using the left prefrontal cortex of the brain tissue of AD patients and controls [[1](#_ENREF_1), [78](#_ENREF_78), [79](#_ENREF_79)]. NA, not available.
